# Revisiting PNS Plasticity: How Uninjured Sensory Afferents Promote Neuropathic Pain

**DOI:** 10.3389/fncel.2020.612982

**Published:** 2020-12-10

**Authors:** Emily L. Tran, LaTasha K. Crawford

**Affiliations:** Department of Pathobiological Sciences, University of Wisconsin-Madison School of Veterinary Medicine, Madison, WI, United States

**Keywords:** sensory ganglia, neuropathic pain, uninjured afferents, dorsal root ganglia (DRG), spinal nerve ligation (SNL), spared nerve injury (SNI), primary afferent, mechanoreceptor

## Abstract

Despite the widespread study of how injured nerves contribute to chronic pain, there are still major gaps in our understanding of pain mechanisms. This is particularly true of pain resulting from nerve injury, or neuropathic pain, wherein tactile or thermal stimuli cause painful responses that are particularly difficult to treat with existing therapies. Curiously, this stimulus-driven pain relies upon intact, uninjured sensory neurons that transmit the signals that are ultimately sensed as painful. Studies that interrogate uninjured neurons in search of cell-specific mechanisms have shown that nerve injury alters intact, uninjured neurons resulting in an activity that drives stimulus-evoked pain. This review of neuropathic pain mechanisms summarizes cell-type-specific pathology of uninjured sensory neurons and the sensory ganglia that house their cell bodies. Uninjured neurons have demonstrated a wide range of molecular and neurophysiologic changes, many of which are distinct from those detected in injured neurons. These intriguing findings include expression of pain-associated molecules, neurophysiological changes that underlie increased excitability, and evidence that intercellular signaling within sensory ganglia alters uninjured neurons. In addition to well-supported findings, this review also discusses potential mechanisms that remain poorly understood in the context of nerve injury. This review highlights key questions that will advance our understanding of the plasticity of sensory neuron subpopulations and clarify the role of uninjured neurons in developing anti-pain therapies.

## Introduction

Neuropathic pain, or pain resulting from nerve injury, can be induced by any range of causes spanning from traumatic to chemical to metabolic insults. For example, a nerve innervating the skin may be severely damaged, eventually resulting in loss of innervation of that target region. However, instead of a numb, insensate phenotype that one might expect to see with a loss of innervation, stimuli applied to the adjacent skin often result in exaggerated or prolonged pain in response to noxious stimuli, termed hyperalgesia, or even painful responses to innocuous stimuli, termed allodynia. Curiously, this stimulus-driven pain relies upon intact primary sensory neurons to conduct the signals that are ultimately interpreted as painful. In conditions where the peripheral terminals of injured neurons die off, how do intact, uninjured sensory neurons go from being spared bystanders of injury to become drivers of neuropathic pain?

This review examines the plasticity of uninjured sensory neurons through the lens of the cell-specific mechanisms of pain. Key studies in sensory biology have unearthed the vast diversity of sensory neurons, also termed primary afferents. In oversimplified terms, primary afferent neurons include several subtypes of mechanoreceptors that encode tactile information, nociceptors that encode painful information, pruriceptors that encode itch, and subsets of neurons that encode a combination of modalities. Sensory ganglia such as the dorsal root ganglion (DRG) house the cell bodies of mixed populations of sensory neurons, each with a pseudounipolar morphology whose axon bifurcates to innervate tissue with the peripheral branch and the spinal cord or brainstem with the central branch. Each sensory neuron is believed to be specifically tuned to particular types of stimuli given a combination of its receptive field, receptors expressed on peripheral axon terminals, axon diameter, intrinsic membrane properties, level of myelination, communication with adjacent glia and other neurons within the DRG, distribution of central axon terminals, and communication between central terminals and spinal cord neurons.

Though central sensitization arising from altered spinal cord circuits is an important contributor to pain mechanisms, it will not be addressed in detail in this review article. However, the central terminals of sensory neurons do deserve some consideration in the context of primary afferent plasticity. The majority of C-fibers terminate primarily at the corresponding level of the spinal cord. However, up to ~25% of C-fiber nociceptors extend terminals one to two segments rostral or caudal to the segment of the entry (Shehab et al., 2008; Olson et al., 2017). This multi-level distribution is even more striking for Aβ low threshold mechanoreceptor (LTMR) subtypes, which send terminals to the deep dorsal horn lamina at that level of the spinal cord, along with shorter caudal collateral and longer rostral collaterals that terminate many segments away from this level. Also, the Aβ LTMR collaterals project directly to the gracile nucleus or cuneate nucleus of the brainstem (Li et al., 2011; Abraira and Ginty, 2013; Niu et al., 2013). Thus increased excitability or spontaneous activity in Aβ afferents could potentially affect pain circuits at that level of the spinal cord along with a wide range of segments of the ipsilateral spinal cord and the ipsilateral brainstem. The large diameter axon and heavy myelination of Aβ LTMRs could provide for more reliable transmission of signals than what you might see for Aδ or C fibers. Finally, the significant overlap of axon terminals from DRGs at adjacent levels of the spine, or even distant levels of the spine in the case of Aβ LTMRs, provide anatomic substrates for changes to other primary afferents and local pain circuits. Subtype-specific mechanistic questions are not just interesting to ponder in the context of uninjured neurons, they are crucial to understanding the role of anatomic, molecular, and neurophysiologic changes in pain behaviors.

## A Synopsis of the Neuropathology of Axotomy

An understanding of the canonical reaction to axotomy can lend insight into mechanisms that may affect even uninjured, bystander neurons after nerve injury. After axotomy, the entire axon from the site of injury to the axon terminals swell and then degenerate in a process termed Wallerian degeneration that has been described in countless reviews and textbooks (Purves, 2012; DeLahunta et al., 2015; Zachary et al., 2017). In brief, secondary to axon degeneration, the Schwann cell processes that enwrap axons to provide the myelin sheath degenerate, leaving their nuclei and a small degree of the cytoplasm of Schwann cells intact. An influx of macrophages phagocytizes necrotic debris distal to the injury. Just proximal to injury the axon undergoes traumatic degeneration, which involves many of the same mediators as Wallerian degeneration distal to the injury. Schwann cells then proliferate, form longitudinal arrays along pre-existing basal lamina, and produce neurotrophic factors that promote axon regeneration. Depending on the nature of the injury, regeneration includes axon sprouting and formation of a neuroma consisting of a haphazard accumulation of growth cones, mature axon terminals, connective tissue, blood vessels, and Schwann cells. After axotomy, this process of regeneration may contribute to ongoing pain (Xie et al., 2017).

Axotomy is accompanied by substantial changes to vasculature that may affect uninjured neurons at the level of axons within the nerve sheath and cell bodies within the DRG. Nerve injury induces traumatic breaks in the endoneurial vessels within the nerve sheath, increased vascular permeability, and leaking of serum contents into the endoneurium that is more severe distal to the lesion (Olsson, 1966). Also, changes are seen at some distance from the site of injury, including increased vascular permeability that contributes to nerve edema (Olsson, 1966) and provides a means for inflammatory mediators released at the site of injury to gain entry into the vascular system. Unlike the blood-brain barrier of the CNS or the blood-nerve barrier of the peripheral nerve, the DRG contains no barrier. DRG blood vessels are lined by a mixture of continuous endothelium and fenestrated endothelium (Arvidson, 1979), the latter being reminiscent of the fenestrated capillaries of endocrine organs and kidney glomeruli. Leakage of protein tracers is also seen in the nerve root and DRG after intravascular injection (Olsson, 1968, 1971), suggesting the nerve root has endothelial permeability comparable to that of the DRG. Although vascular changes within the DRG have not been thoroughly explored in models of neuropathic pain, injury-induced vascular changes within the DRG may include changes seen in other tissues after localized injury, including endothelial hypertrophy, changes in the membrane expression of molecular markers, dysfunctional endothelial cell-cell adhesion, and increased vascular permeability. Some evidence of this includes an increase in PI16 in fibroblasts in DRG meninges and in the perineurial space of the sciatic nerve after injury, which leads to increased leukocyte infiltration in DRG and promotes pain behaviors in mice (Singhmar et al., 2020). Interestingly, isolectin B4 (IB4) binding to vascular endothelium has also been noted in the DRG after nerve injury (Li and Zhou, 2001), and it has been reported as a marker of injury-induced neovascularization in the spinal cord and eye (Benton et al., 2008; Mahoney et al., 2009; Walchli et al., 2015).

Throughout this reaction to nerve injury, uninjured neurons are potentially affected at several locations. Intact axons could be exposed to the cytokines, chemokines, inflammatory mediators, and neurotrophic factors released from necrotic axons, infiltrating inflammatory cells, reactive endothelium, and proliferating Schwann cells. These mechanisms would be particularly relevant for uninjured neurons whose axons travel within the same nerve sheath, directly adjacent to injured axons. Also, cytokines, chemokines, growth factors, and other mediators released at the site of injury can cross into the bloodstream *via* the endothelium that is disrupted by injury and ensuing inflammation. The lack of a blood barrier around the DRG paired with further increases in vascular permeability after injury could contribute to increased delivery of these circulating mediators to DRG neurons and satellite glia, thus altering neuronal excitability or priming for activation by other pathways. In fact, after nerve injury, coincident exposure of cytokines, chemical modulators, and other neuronal mechanisms are thought to contribute to the persistence of pain (Matsuka et al., 2020).

## Methods That Identify Uninjured Neurons in Models of Neuropathic Pain

The study of cell-specific mechanisms of neuropathic pain has generated several rodent models that distinguish between uninjured neurons to clarify distinct mechanisms of plasticity and dysregulation. The spinal nerve ligation (SNL) model is typically characterized in mice by ligation of the L4 spinal nerve just distal to the L4 DRG, affecting many fibers that innervate the distal hind limb (Kim and Chung, 1992; Kim et al., 1997). While all neurons in the ipsilateral L4 DRG are injured, the adjacent L3 DRG consists entirely of uninjured neurons. Concerning L4 axons, some uninjured L3 axons course alongside axons from injured L4 neurons within the sciatic nerve sheath, L3 peripheral terminals innervate overlapping regions of the skin, and L3 central terminals overlap within the spinal cord dorsal horn. SNL studies in rats typically use ligation of the L5 nerve and selectively analyze L4 and L3 DRG to target uninjured neurons. In both mice and rats, investigators may cut the spinal nerve distal to the ligation or instead of ligation, a model sometimes referred to as modified SNL or spinal nerve axotomy (SNA; Li et al., 2000; Djouhri et al., 2006).

The Spared Nerve Injury (SNI) model (Decosterd and Woolf, 2000), the Selzer model (Shir and Seltzer, 1990), and variations of these nerve injury models selectively ligate and/or axotomize part of the sciatic nerve or branches of the sciatic nerve distal to its trifurcation, leaving an intact proportion of the sciatic nerve bundle or an intact branch of the sciatic nerve. In mouse SNI models that leave the sural branch of the sciatic nerve uninjured or “spared,” injured and uninjured neurons are intermingled with the L3, L4, and L5 DRGs wherein L3 and L4 have a higher proportion of injured neurons than L5, and uninjured neurons are most abundant in L4 and L5 (Laedermann et al., 2014). A similar mix of injured and uninjured neurons are seen in the L4, L5, and L6 DRGs of rats following SNI (Decosterd and Woolf, 2000). Uninjured neurons have been distinguished in these models using a post-surgical injection of retrograde tracers in the target area of the skin, wherein uptake and transportation to the cell body are only possible by intact axons of uninjured neurons (Ma and Bisby, 1997; Hudson et al., 2001). Another variation is to perform another surgery to reveal the distal aspect of the intact nerve and inject tracer directly into the nerve (Decosterd et al., 2002). This appears to increase the labeling efficiency of intact neurons, with the caveat that intraneural injection of the tracer may itself cause some degree of injury to otherwise intact neurons. Another method to identify uninjured neurons is to analyze neurons that lack markers of neuronal injury, such as the transcription factor ATF3. However, this method alone is not very sensitive, as it does not discriminate between uninjured neurons that innervate hypersensitive skin and neurons that innervate distant, unrelated parts of the body at that level of the spine. Also, some studies have shown sparse ATF3 labeling in non-axotomized, uninjured neurons after surgical exposure, as might occur in a sham procedure or in nerves that are adjacent to the nerves targeted for surgical injury (Fukuoka et al., 2012). Thus, identifying uninjured neurons by the absence of injury-markers lacks both sensitivity and specificity, though it may provide a degree of insight about mechanisms that can be elucidated further with more precise methods.

## Altered Expression of Molecular Markers in Uninjured Neurons After Injury

**Figure 1:**
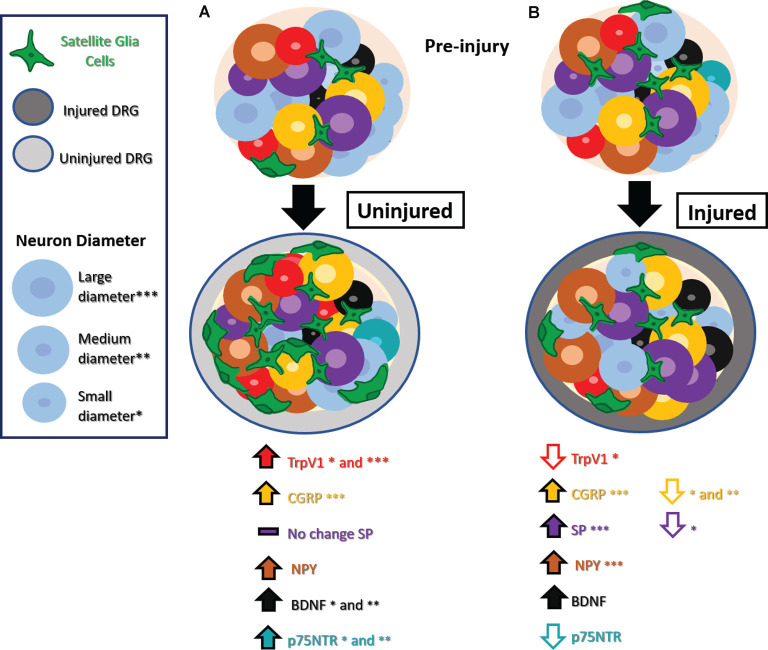
Phenotype switching and other molecular changes in uninjured sensory neurons after nerve injury. **(A)** Uninjured dorsal root ganglion (DRG) neurons exhibit increased TrpV1 in small and medium-diameter neurons, CGRP in large-diameter neurons, NPY, BDNF in small- and medium-diameter neurons, and no significant changes in SP. Small- and medium-diameter neurons display increases in p75NTR as well. There is also an increase in satellite glial cells (SGCs). **(B)** Injured DRG neurons show decreased TrpV1 in small-diameter neurons, decreased CGRP in small- and medium-diameter neurons decreased SP in small-diameter neurons, and decreased p75NTR. Injured DRG neurons show increased BDNF, and injured large-diameter neurons show increased CGRP, SP, and NPY. An increase in SGCs appears in injured DRG as well. Abbreviations: TrpV1, transient receptor potential vanilloid 1; CGRP, calcitonin gene-regulated peptide; SP, substance P; NPY, neuropeptide Y; BDNF, Brain-derived neurotrophic factor; p75NTR, neurotrophin receptor p75. Asterisks denote cell diameter size, the absence of these indicate no reported differences between cells of different diameters.

A range of studies has explored changes in expression in nociceptive markers in the DRG after injury. It has been proposed that these markers are expressed de novo by non-nociceptive neurons after nerve injury, a phenomenon known as phenotype switching. The emerging hypothesis is that phenotype switching may provide a mechanism whereby innocuous mechanical stimuli that activate non-nociceptive neurons well-tuned to that type of stimulus now trigger the release of nociceptive mediators, contributing to hypersensitive and allodynia pain-like behaviors. With increasing attention to uninjured neurons, a range of other unique molecular changes has been noted in uninjured neurons, as summarized in Figure 1.

### Transient Receptor Potential Cation Channel Vanilloid Type 1 (TrpV1)

TrpV1, the receptor responsible for sensing the chemical compound capsaicin, is typically expressed by small diameter C-fiber nociceptors. However, after nerve injury, TrpV1 expression increases in the DRG and is expressed ectopically by other types of neurons. TrpV1 expression in the rat has been examined after several types of nerve injury, where it shows a decreased expression in injured neurons after sciatic axotomy, partial transection, and SNL (Hudson et al., 2001). However increased TrpV1 expression was seen in uninjured neurons, including small diameter C fibers, along with ectopic expression in large diameter-A fibers (Hudson et al., 2001). The contribution of uninjured DRG to stimulus-evoked pain is underscored by the fact that chemical ablation of TrpV1-expressing neurons of uninjured L3 and L4 nerves could completely alleviate thermal hyperalgesia and mechanical allodynia after L5 SNL (Javed et al., 2020).

### Calcitonin Gene-Related Peptide (CGRP)

CGRP, known as a marker of peptidergic nociceptors, exhibits marked changes in distinct subpopulations after nerve injury. At the mRNA level, L5 SNL in the rat results in an increase in CGRP mRNA in uninjured L4 neurons and a decrease in CGRP mRNA in injured L5 neurons, compared to the respective contralateral DRG (Fukuoka et al., 1998). In this study, this increase in uninjured L4 neurons was interpreted to be due to the upregulation of native CGRP expression rather than *de novo* expression in larger neurons. Studies that carefully distinguish neuronal subsets and spinal cord lamina have demonstrated that small and medium diameter injured L5 neurons show a decrease in CGRP protein in their cell soma and a decrease in CGRP-labeled terminals in the superficial nociceptor layer of the ipsilateral spinal cord (Nitzan-Luques et al., 2011). However, a distinct picture emerges when looking at large diameter neurons. In a study of SNL in rats, there was an increase in CGRP protein in injured large-diameter L5 DRG neurons, particularly in those rats that exhibit the most severe pain behaviors (Nitzan-Luques et al., 2011, 2013). The increased CGRP in injured large-diameter neurons was accompanied by an increase in CGRP-labeled terminals in the deep lamina of the dorsal horn as well as ectopic CGRP expression in the gracile nucleus, sites that receive the central terminals of Aβ low threshold mechanoreceptors (LTMRs; Nitzan-Luques et al., 2011, 2013). Pharmacologic blockade paired with selective activation of distinct fiber types further implicates CGRP expression in A fiber LTMRs as a major contributor to allodynia pain behaviors after nerve injury (Nitzan-Luques et al., 2013). The diverging findings between these CGRP studies have not been reconciled but could be due to distinctions in regulation at the mRNA vs. protein levels, or genetic differences in the strain of rats used. Another possible explanation could be the distinct controls used for comparisons in these studies. Studies that lack sham or naive controls cannot rule out the possible confound of bilateral changes that may obfuscate comparisons.

### Substance P (SP)

Another nociceptive marker, substance P, is reported to have no change in uninjured neurons after SNL, but a long-lasting increase in injured large-diameter neurons (Weissner et al., 2006). The expression of Substance P is more dynamic with a transient increase followed by a longstanding decrease in injured small-diameter neurons (Weissner et al., 2006). The significance of this upregulation is unclear, as some studies have shown that Substance P does not seem crucial for developing tactile allodynia after SNL in rats (Hughes et al., 2007; Nitzan-Luques et al., 2011).

### Neuropeptide Y (NPY)

NPY is not expressed in the DRG of naïve rodents but is highly upregulated in primarily large-diameter injured neurons after nerve injury (Wakisaka et al., 1991; Fukuoka et al., 1998; Hokfelt et al., 1998; Ma and Bisby, 2000; Nitzan-Luques et al., 2011) Interestingly, this increase is negatively correlated to the severity of heat hyperalgesia in a chronic constriction injury (CCI) model of nerve injury, suggesting it may serve a protective role rather than contribute to pain behavior (Ruscheweyh et al., 2007). Additional studies have identified the analgesic effects of NPY, acting *via* NPY receptors Y1 and Y2 in the dorsal horn of the spinal cord in the face of neuropathic pain, along with other forms of pain (Kuphal et al., 2008; Solway et al., 2011). In contrast, studies by others have shown that intracisternal administration of a Y1 receptor antagonist dampens allodynia after SNL, suggesting that NPY receptors may contribute to pain (Fukuoka and Noguchi, 2015).

### Brain-Derived Neurotrophic Factor (BDNF)

Brain-derived neurotrophic factor (BDNF) is a modulator of neuronal plasticity that is thought to be pro-nociceptive (for review see Garraway and Huie, 2016). BDNF has been noted to increase at the mRNA and protein level in ATF3-negative, uninjured small, and medium diameter neurons after SNL in the rat (Fukuoka et al., 2001, 2012; Obata et al., 2003) as well as injured neurons (Michael et al., 1999). Intense primary afferent activity is hypothesized to lead to BDNF release, which could potentiate synaptic plasticity in the spinal cord and contribute to central-sensitization mechanisms (Garraway and Huie, 2016), prime synaptic partners for the effects of co-transmitters such as substance P (Chen et al., 2014), or alter ion channel activity (Cao et al., 2012). BDNF is sexually dimorphic and may play a distinct role in sensation in naïve males vs. females (Liu et al., 2012; Dembo et al., 2018); however, it remains unclear whether the role of BDNF in uninjured neurons differs between males and females after nerve injury.

### Nerve Growth Factor (NGF) and p75 Neurotrophin Receptor (p75NTR)

Another neurotrophic factor that has garnered a lot of attention is nerve growth factor (NGF) and its receptor, the p75 neurotrophin receptor (p75NTR). After L5 SNL injury, p75NTR is upregulated in small and medium diameter uninjured L4 DRG neurons while it is decreased in injured DRGs neurons (Obata et al., 2006). NGF binds p75NTR and, through the p38 mitogen-activated protein (MAP) kinase pathway, increases expression of BDNF, TrpV1, and TrpA1 contributing to heat and cold hyperalgesia (Obata et al., 2004, 2006). The crucial role of this pathway is underscored by the fact that knockdown of p75NTR expression or pharmacologic blockade of the downstream MAP kinase pathway likewise dampened thermal and cold hyperalgesia as well as mechanical allodynia (Obata et al., 2004, 2006). Anti-NGF antibody applied to the uninjured L4 nerve or intrathecally near the lumbar DRG can dampen thermal and cold hyperalgesia (Obata et al., 2004). Anti-NGF antibody applied intrathecally near the lumbar DRG inhibits NGF upregulation in satellite glia of contralateral DRG and dampens “mirror-image” pain on the contralateral limb (Cheng et al., 2014), implicating activated DRG satellite glia as a potential source of excess NGF in nerve injury conditions, in addition to the injured nerve itself (Fukuoka et al., 2001).

### Emerging Molecular Markers of Uninjured Neurons

Advances in high-throughput and unbiased RNA sequencing technologies have allowed for the identification of molecules and pathways that are newly implicated in neuropathic pain. Studies looking at injury-induced changes in gene expression in DRG suggest that there is a wide breadth of molecules that become up or downregulated. Some common molecules that appear upregulated after injury across several studies include ATF3 and NPY (Hammer et al., 2010; Hu et al., 2016; Wu et al., 2016; Sun et al., 2020) as well as inflammatory molecules like IL-6 (Gong et al., 2016; Chen et al., 2017) and other key genes in immune cell signaling pathways like CD74 (Sun et al., 2020) and CDK6 (Chen et al., 2017). Most of these studies do not look at uninjured DRG specifically but instead pool together uninjured and injured DRG from injured animals. Whether uninjured and injured DRG show similar or divergent changes at the RNA level warrants further attention. Recent evidence suggests that, while some molecular changes are the same, uninjured and injured DRG do have some distinct changes in gene expression (Chen et al., 2017). In this study, L5 SNL in rats leads to increased expression of molecules associated with cell adhesion, such as Ctnnb1 which encodes β-catenin and was upregulated in both injured and uninjured DRG neurons. Changes that were unique to uninjured neurons included downregulation of Ahcyl1 and upregulation of Eif4a2 and Wt1, genes that encode molecules involved in signaling pathways, transcription, or tumor development but that are relatively unexplored in the context of neuropathic pain (Chen et al., 2017).

Few studies have performed unbiased transcriptomics evaluating specific subtypes of uninjured neurons. One study that targeted nociceptors at SNI day 7 identified an increase in caspase-6 in pooled samples of injured small-diameter neurons but no changes in pooled samples of uninjured small-diameter neurons (Berta et al., 2017). Single-cell technologies are producing data with a more precise lens, showing that when evaluating only injured neurons after sciatic transection, different neuronal subtypes exhibit distinct changes after injury in mice (Hu et al., 2016). Single-cell transcriptomics will be a useful tool to examine uninjured neurons in more detail to determine new molecular changes in distinct subtypes of uninjured neurons, particularly large diameter uninjured neurons such as various Aβ LTMRs that have molecular markers that can be identified in single-cell studies (Usoskin et al., 2015).

### Potential Implications of Phenotype Switching

Although direct evidence for the consequences of phenotype switching is not always available, the implication is that attaining de novo expression of one of the above markers renders that neuron capable of releasing a different type of neurotransmitter, conveying a different type of message. *De novo* expression of nociceptive peptides in mechanoreceptors may lead to the release of nociceptive peptides from their terminals in the ectopic, deep lamina of the spinal cord. In the case of Aβ LTMRs, this could change the neurotransmitter released at multiple spinal cord segments as well as within the gracile nucleus. Phenotype switching could also alter what is released within the DRG and what is released from sensory neuron axon terminals in innervated regions of the skin. Finally, phenotype switching can likewise alter which neurons are susceptible to pharmacologic agents that target these receptors, neurotransmitters, and pathways. As our field develops a more nuanced understanding, these phenotype switching mechanisms may provide an avenue to modulate targeted subsets of sensory neurons that emerge in pathologic conditions.

## Altered Neurophysiology of Uninjured Neurons After Injury

Early studies that characterized nerve injury models showed that uninjured nerves can demonstrate spontaneous activity at sites that are some distance from nerve injury (Baik-Han et al., 1990) and those uninjured nerves are particularly important for evoked pain (Yoon et al., 1996). Examination of primary afferent neurophysiology has revealed interesting mechanisms that underlie excitability and spontaneous activity of uninjured neurons following nerve injury. Intact L4 neurons have demonstrated altered intrinsic membrane properties that contribute to increased excitability along with increased spontaneous firing after L5 SNA. These effects were even more prominent in a modified-SNA (mSNA) variant of the model wherein intact L4 nerve fibers conduct through a nerve partially damaged by loose ligation with chromic gut (Djouhri et al., 2012). The spontaneous and stimulus-driven pain behaviors of mSNA rats were likewise more severe than SNA rats, suggesting that the increased excitability and spontaneous activity of uninjured neurons in the mSNA model contribute to increased severity of pain behaviors (Djouhri et al., 2012). A growing number of studies have examined how the altered neurophysiology of specific subtypes of uninjured neurons contributes to neuropathic pain as detailed below and summarized in Table 1.

**Table 1:** Neurophysiologic changes in uninjured sensory neurons after nerve injury.

C fibers	Aδ fibers	Aβ fibers	Model of Neuropathic pain	References
↑ Nav1.8 mRNA uninjured C ↑ TTx-Rz current uninjured C ↓ Nav1.8 mRNA injured C ↓ TTx-Rz current injured C			SNL	Zhang et al. (2004)
↓ Mech threshold uninjured C ↑ Adrenergic sensitivity uninjured C ↑ Spont firing uninjured C			SNL (monkey)	Ali et al. (1999)
↑ Spont firing uninjured C		↓ Mech threshold uninjured Aβ nociceptor ↑ Responsiveness to mech stimuli in uninjured Aβ nociceptor	SNA, mSNA, CFA	Djouhri et al. (2006)
↑ Spont firing uninjured C	↑ Spont firing uninjured Aδ	↑ Spont firing uninjured Aβ nociceptor ↑ Spont firing uninjured Aα/β LTMR	SNA, mSNA	Djouhri et al. (2012)
↑ I-h current in uninjured C ↑ Excitability in uninjured C ↑ Spont firing in uninjured C		↑ Spont firing uninjured Aβ LTMR, independent of I-h current	mSNA	Djouhri et al. (2018)
No change spont firing uninjured C	↓ Rheobase uninjured and injured A-fibers ↑ Spont firing uninjured A fibers and injured A-fibers		SNL, rhizotomy	Ma et al. (2003)
IL-β leads to ↑ Cav2.2 ↓ Rheobase ↑ Excitability uninjured neurons (multiple fiber types)			SNL	Yang et al. (2018)
Cav2.2 blocker ↓ Uninjured excitability and ↓ pain (multiple fiber types) IL-10 leads to ↓ Cav2.2 in injured neurons (multiple fiber types)		

*Abbreviations: ↑, increase; ↓, decrease; mech, mechanical; spont, spontaneous; RF, receptive field; TTx-Rz, tetrodotoxin-resistant. Abbreviations for neuropathic pain models are defined in the main text and refer to rodent models, except where noted*.

### Excitability and Spontaneous Activity in C-fibers

Looking more closely at neuronal subtypes, several studies have demonstrated that intact uninjured C-fibers show spontaneous activity after nerve injury (Ali et al., 1999; Wu et al., 2001; Shim et al., 2005; Djouhri et al., 2012) and this increased spontaneous firing correlates to the severity of spontaneous pain behaviors (Djouhri et al., 2006; Chen et al., 2019) and contributes to evoked thermal hyperalgesia (Shim et al., 2005). After mSNA C nociceptors were noted to have an increased I-h current in intact L4 DRG. This increased I-h current resulted in hyperexcitability and spontaneous activity in uninjured C nociceptors, which was thought to contribute to cold allodynia, spontaneous pain, and mechanical allodynia (Djouhri et al., 2018). Both Nav1.7 and Nav1.8 channels can contribute to nociceptor activity and have been implicated in pain syndromes in humans (McDermott et al., 2019). After SNL, uninjured neurons show an increase in mRNA and protein levels of Nav1.7 and Nav1.8 thought to contribute increases in spontaneous activity (Zhang et al., 2004; Li et al., 2019). While other studies have reported no change in Nav1.7 at the level of mRNA after SNL (Fukuoka et al., 2012), a decrease in Nav1.7 protein and mRNA expression has been reported in injured neurons (Li et al., 2019). After L6 SNL in monkey, injured and uninjured C-fibers showed increased spontaneous activity along with increased α-adrenergic sensitivity in uninjured nerve fibers (Ali et al., 1999).

### Excitability and Spontaneous Activity in Various Subtypes Including A-fibers

Additional studies have expanded their focus to include other types of sensory neurons. Single fiber recordings of peripheral nerves in rats after L5 and L6 SNL demonstrated a lower mechanical threshold in uninjured C and Aδ fibers that may underlie the increased mechanical sensitivity exhibited by those animals (Shim et al., 2005). Uninjured A-fibers also show neurophysiological changes and spontaneous activity after nerve injury. Intact L4 Aβ LTMRs showed spontaneous activity after mSNA, though the mechanism is independent of I-h channels, as their spontaneous activity was not affected by the I-h channel blockade (Djouhri et al., 2018). *In vivo* electrophysiology studies that targeted multiple sensory fiber subtypes have identified spontaneous firing in uninjured L4 C-, Aδ-, and Aβ-nociceptors and cutaneous Aα/βLTMRs after nerve injury (Djouhri et al., 2012). In another study, Ma et al. (2003) identified lower rheobase and increased spontaneous activity in uninjured myelinated (A-fiber) neurons with no change in uninjured C-fibers. Studies in rats using L5 SNL have demonstrated an increase in Cav2.2 expression that contributes to hyperexcitability and lower rheobase in uninjured L4 neurons (Yang et al., 2018). The underlying mechanism was thought to be due to an increase in local levels of IL-1β in the uninjured L4 DRG, which increases the expression of Cav2.2. In injured DRG, increased levels of IL-10 were thought to lead to decreased Cav2.2 in injured neurons. Cav3.2 expression also changes after injury, possibly increasing within uninjured peripheral nerve fibers and contributing to increased excitability and lower mechanical threshold in a subset of C and Aδ fibers (Chen et al., 2018).

After L5 SNL, others have shown uninjured L4 Aδ fibers that innervate skin have a decreased mechanical threshold, which was not seen in the corresponding C-fibers (Ji et al., 2007). This study also characterized an increase in the number of Aδ neurons that were sensitive to cold or to the TrpM8-agonist icilin, along with an increased icilin response amplitude. This was accompanied by an increase in the number of C-mechano-cold-fibers, but no change in C-fiber icilin responses.

Other studies using the partial SNL model applied to L5 in rats have shown uninjured L4 A-fiber high threshold mechanoreceptors (A-HTMRs) have decreased mechanical threshold, increased receptive field area, faster AP rise times, small AHP amplitude, and increased excitability, while uninjured C-fibers have decreased mechanical threshold with no intrinsic membrane changes detected *via* *in vivo* intracellular electrophysiology (Boada et al., 2015). Conversely, A-fiber LTMRs were desensitized, demonstrating increased mechanical threshold, slower AP rise times, decreased receptive field area, and decreased excitability. Interestingly, it appears that the mechanical threshold of high-threshold and low threshold mechanosensitive neurons completely overlapped in this study, as did the size of their receptive fields and their maximum excitability in response to a mechanical stimulus. Also, following injury, there was a larger proportion of neurons that were not excitable, increased numbers of LTMRs that could not be categorized into a specific fiber type, and an increased number of neurons for which the receptive field could not be identified, and whose active properties were intermediate between HTMRs and LTMRs (Boada et al., 2015). These findings suggest that dividing LTMRs from HTMRs and distinguishing LTMR subtypes may be difficult after nerve injury if relying solely upon neurophysiological properties that are, themselves, altered by the pathology.

### Myelination and Conduction Velocity

Neurotransmission of sensory information is also dependent upon proper myelination of myelinated fibers. Induced demyelination of whole nerve or selective demyelination of A-fibers can lead to pain (Wallace et al., 2003; Inoue et al., 2004; Ahn et al., 2009; Zhu et al., 2012b; Duan and Xie, 2016). While evidence of demyelination is not often noted in surgical models of nerve injury, it should be noted that demyelination is often examined without attention to distinctions between injured and uninjured fibers or fiber subtypes. In nerve injury models, uninjured L4 C-fibers show more activity-induced slowing of conduction velocity after L5 SNL (Shim et al., 2007), and an unspecified mix of injured and uninjured low threshold A-β fibers show decreased conduction velocity in a CCI-like model of inflammatory nerve injury (Zhu et al., 2012a). Although these studies attributed changes in conduction velocity to altered HCN channels, cell-specific changes in myelination were not directly addressed. Data from other studies often suggest an increase in the variability of conduction velocity, an increase in the number of “uncategorized neurons” that could not be categorized based on conduction velocity, increased conduction failure, or increased numbers of non-responsive neurons (Boada et al., 2015; Wang et al., 2016). These findings present intriguing reasons to take a closer look at myelination with cell-type-specific precision following nerve injury.

### Implications of Altered Neurophysiology

Ultimately, hyperexcitable neurons may show larger responses to low-intensity input, potentially contributing to hypersensitive behavioral responses. Spontaneous activity in the absence of stimuli, also termed ectopic discharge, is thought to contribute to ongoing pain behaviors such that spontaneous firing in nociceptors contributes to spontaneous pain that persists in the absence of a specific stimulus. Ectopic discharge in DRG is a primary driver of tactile allodynia after SNL in rats, more so than discharge at the site of injury or in the intact axons found in nerves (Yatziv and Devor, 2019). Although spontaneous firing in LTMRs is thought to contribute to dysesthesias or paraesthesias, mechanoreceptor contributions to spontaneous pain are less clear. Both mechanical and nociceptive input can contribute to the sensitization of spinal cord circuits, termed central sensitization (Chung and Chung, 2002; Sukhotinsky et al., 2004). Increased activity of C-fibers can drive innate pain circuits through synapses in the superficial dorsal horn. The increased activity of A-fibers is likewise important for pain, yet these effects may be more nuanced, given the diverse roles that A-fiber central terminals can play. Increased activity in Aβ afferents can drive pain pathways due to de novo expression of pain-related neuropeptides and ion channels and the ability to trigger central sensitization (Devor, 2009). However, Aβ neurons have also been shown to drive inhibitory spinal cord interneurons thereby inhibiting pain signals, as delineated in the spinal cord Gate Control Theory (Braz et al., 2014). It is possible that in some conditions, increased activity of Aβ LTMRs may dampen nociceptive inputs. Finally, Aβ LTMRs have a unique distributed projection pattern such that lumber Aβ LTMRs have terminals at many levels of the spinal cord and in the brainstem (Li et al., 2011). Thus, increased activity in lumbar Aβ LTMRs could either sensitize or dampen localized pain circuits at the lumbar spinal cord, or dispersed pain circuits at other levels of the spinal cord and brainstem. We are just beginning to understand the effect of injury upon Aβ input to the spinal cord. The work by some suggests there is a loss of Aβ-induced inhibition of nociceptive pathways after SNL, such that Aβ inputs can more directly excite nociceptive pathways, therein contributing to allodynia (Lu et al., 2013). However, it is unclear whether other projections of Aβ terminals are altered by nerve injury, and whether pain circuits centered in other segments of the body would be sensitized or dampened as a result.

## Plasticity Within the DRG

### Injury-Induced Intra-ganglionic Plasticity

As we increase our understanding of the molecular and cellular mechanisms of plasticity, we are learning more about the intra and intercellular communication that occurs within the DRG, which is somewhat unique from that seen in the central nervous system. Neuronal intercellular communication between DRG neurons includes neurotransmission and electrical coupling *via* gap junctions between neurons and satellite glial cells, as reviewed most recently by Hanani and Spray (Pannese et al., 2003; Dublin and Hanani, 2007; Hanani and Spray, 2020). An increase in gap-junction mediated electrical coupling between hyperexcitable neurons and satellite glial cells has been shown after axotomy (Cherkas et al., 2004) and CCI (Kim et al., 2016). In addition to neuron-glia interactions, increased glia-glia coupling is likewise associated with chemotherapy-induced neuropathic pain (Warwick and Hanani, 2013). An emerging hypothesis proposes that changes in neural excitation activate surrounding glia and cause them to release cytokines and other signaling molecules that contribute to pain (Matsuka et al., 2020). These studies show heterogeneity in the pattern of coupling that includes a subset of Aβ LTMRs and TrpV1-positive nociceptors, yet it remains unclear whether this coupling involves injured neurons, uninjured neurons, or a mixture of both.

Other injury-induced changes include the formation of scattered ring-like structures that contain satellite glial cells, IB4-positive neuronal processes, macrophages, and TH-positive axons postulated to extend from sympathetic nerves (Li and Zhou, 2001; Vega-Avelaira et al., 2009). Notably, these ring structures are predominantly centered on large diameter A-fiber neurons. Sympathetic axons, driven by satellite cell-derived growth factors, extend from DRG vasculature and preferentially surround neurons that project to the gracile nucleus (Ma and Bisby, 1999; Zhou et al., 1999), i.e., Aβ LTMRs. They also tend to surround spontaneously active neurons (Xie et al., 2011), though other studies show comparable targeting of injured and uninjured neurons (Ma and Bisby, 1999). It remains unclear whether there is selective localization of IB4 neuronal processes or microglia/macrophages around injured or uninjured subpopulations. The signals directing the selective localization of these structures, their effects on neuronal activity, and their potential contribution to pain mechanisms remain poorly understood.

Collectively these studies suggest that intraganglionic communication is an important contributor to neuropathic pain. It will be interesting to learn if uninjured neurons housed within the same DRG as injured neurons exhibit distinct changes compared to uninjured neurons found in DRGs at other levels of the spine or in contralateral DRGs. These intricacies highlight the fact that, although popular for molecular and neurophysiology studies, dissociated DRG culture is limited in its ability to recapitulate DRG circuits. Furthermore, it has been shown that dissociated cell culture alters gene expression of DRG neurons in both mouse and human, including decreased expression of G-protein-coupled receptors and ion channels and increased expression of injury-associated markers such as BDNF and ATF3 amongst others (Wangzhou et al., 2020). Studies that do not rely upon destroying connections between neurons and glia and inducing acute injury to the neurons will be crucial for advancing our understanding of DRG physiology.

### Possible Mechanisms Unexplored in the Context of Nerve Injury

Other facets of the unique neurophysiology of DRG neurons include GABA signaling and the inverted chloride gradient that help mediate the effects of GABAergic neurotransmission. The synthetic enzymes for GABA and its receptors are widely expressed amongst DRG neurons at the mRNA and protein level (Hanack et al., 2015; Du et al., 2017) and both electrical stimulation of DRG neurons and local exogenous GABA evoke GABA(A) receptor-mediated currents in DRG neurons (Zhu et al., 2014; Du et al., 2017). These elements help form what some have termed a DRG gate, or localized inhibitory circuit consisting of GABAergic DRG neurons, capable of inhibiting the transmission of pain signals in naïve animals, after inflammatory insult, and after induction of the CCI inflammatory nerve injury model. Although activating this DRG “brake” can dampen painful insults, it is unknown if dysfunction of this brake contributes to the pain after nerve injury. One study identified a decrease in GABA(A) R gamma2 subunit in injured L5 neurons compared to a contralateral control, with no changes noted in uninjured L4 DRGs (Fukuoka et al., 1998). However, cell-specific effects of injury have not been thoroughly explored across other components of GABA signaling within the DRG.

Another key component to understanding DRG GABA circuits is the chloride gradient. Under healthy conditions, the high expression of NKCC1 and KCC2 channels in DRG neurons increases the intracellular chloride concentration, increasing the reversal potential of GABA(A) chloride channels, like the GABA(A) receptor, up to 30 mV above resting membrane potential (Price et al., 2009). Activation of GABA(A) receptors causes chloride to flow outward, depolarizing the neuron, though this can still be inhibitory, serving as a “shunt.” Bicarbonate-chloride anion exchange channels also play a role. The ability to fire action potentials in the presence of GABA is therefore dependent upon the physiology and relative abundance of GABA receptors and various chloride channels, and these may differ across distinct types of neurons after injury. Interestingly, satellite glial cells could also be implicated in these mechanisms, as they are reported to have the ability to store GABA (Matsuka et al., 2020). Cell-specific changes in these chloride channels have not been thoroughly explored in the context of neuropathic pain. It remains unclear whether ion gradients and GABA signaling, are differentially regulated in injured and uninjured neurons, whether that alters intercellular signaling within the DRG, and how that contributes to neuropathic pain.

Historically, the majority of pain research has occurred in male rodents or in studies that are not statistically powered to detect sex differences. Thus, there are still significant gaps in what we know about sex differences in neuropathic pain mechanisms; this is especially true of uninjured neurons. Some insight into potential sex differences has come from transcriptomic studies of sex differences in the CCI model of inflammatory nerve injury. Shared changes seen in rats of both sexes included upregulated gene expression of ATF3 and VIP (Stephens et al., 2019), which have also been implicated in neuropathic pain. Amongst a wide array of sex differences, male rats exhibited more significant changes in several ion channel genes while female rats expressed changes relating more to synaptic transmission (Stephens et al., 2019). It remains unclear whether sexually dimorphic changes are seen in primary afferents in models of neuropathic pain, whether there are sex differences in the molecular markers or neuroplasticity of uninjured neurons, and how these changes may contribute to differences in pain-related behaviors.

## Discussion

In summary, there are several mechanisms by which uninjured neurons contribute to chronic neuropathic pain, some more well understood than others, especially in the context of nerve injury. These include molecular changes in the type and amount of neurotransmitters produced, the ion channels that mediate neurophysiological properties, and the gap junctions that mediate intraganglionic communication, in addition to several other potential mechanisms. A notable trend in discerning studies is that uninjured and injured neurons often have divergent changes. Further, uninjured mechanoreceptors are frequently implicated in such alterations, quite often with distinctive changes opposite to those seen in nociceptors or that are absent in nociceptors altogether. These are all changes that can be obfuscated in studies that combine injured and uninjured neurons into a single group. A mechanistic understanding of plasticity in uninjured neurons may shed light on the changes that occur contralateral to injury as well. For example, exposure to mediators in the systemic circulation or alterations in descending signals from central circuits may contribute to changes in contralateral, uninjured neurons after injury, as has been suggested of NGF in the cerebrospinal fluid (Cheng et al., 2014). This understanding opens the door to new hypotheses regarding uninjured afferents that innervate other regions of the body. With our current grasp on the neuroplasticity of sensory neurons, several intriguing questions remain:

- What are the mechanisms that underlie alterations in uninjured neurons?
- Do emerging molecular markers that are upregulated in uninjured neurons contribute to pain phenotypes? Does this role differ between females and males?
- Do neuronal-glial interactions differ for uninjured neurons vs. injured neurons?
- Do inflammatory mediators and other signaling molecules have a selective effect on uninjured neurons? If so, is it due to anatomic features of the microenvironment, intracellular pathways that have been primed by evoked activity, or other potential mechanisms?
- Do the peripheral axon terminals of uninjured neurons show changes that parallel the phenotype switching seen in the cell body? After nerve injury can mechanoreceptors release ectopically expressed peptides in response to mechanical stimulation?
- Can we improve the diagnosis of neuropathic pain or track the therapeutic effects of anti-pain therapies using pain-associated markers of uninjured afferents?
- Can receptor agonists or receptor-mediated ablation strategies be used to target uninjured neurons that show ectopic expression of the targeted receptor? Can selective targeting of uninjured neurons help to treat secondary hyperalgesia or allodynia?

Addressing the gaps in our knowledge of primary afferent plasticity and dysfunction will be crucial in propelling the study of neuropathic pain and generating novel diagnostic and therapeutic strategies. This will yield insight into nuanced mechanisms of pain and endogenous analgesia, highlight novel endpoints that can improve the diagnosis of distinct types of pain, and develop interventions that can treat specific modalities of pain as well as complex pain syndromes. Novel strategies can take advantage of the fact that DRGs sit outside of the blood-brain-barrier with their axons sitting in peripheral tissues, which yield pharmacologic access to molecular targets and anatomic access to peripheral devices that are unparalleled by strategies that rely on the central nervous system. Enhanced understanding of how both injured and uninjured neurons are altered in pain states can also potentially prevent unintended off-target side effects or identify opportunities for protective adjunct therapies to strengthen the benefit of existing therapies.

## Author Contributions

LC developed the concept of the manuscript. ET and LC conducted the analysis of the literature and then composed and edited the manuscript text, figures, and tables. All authors contributed to the article and approved the submitted version.

## Conflict of Interest

The authors declare that the research was conducted in the absence of any commercial or financial relationships that could be construed as a potential conflict of interest.
